# Nonprenylated Xanthones from *Gentiana lutea*, *Frasera caroliniensis*, and *Centaurium erythraea* as Novel Inhibitors of Vascular Smooth Muscle Cell Proliferation

**DOI:** 10.3390/molecules201119703

**Published:** 2015-11-13

**Authors:** Birgit Waltenberger, Rongxia Liu, Atanas G. Atanasov, Stefan Schwaiger, Elke H. Heiss, Verena M. Dirsch, Hermann Stuppner

**Affiliations:** 1 Institute of Pharmacy/Pharmacognosy, University of Innsbruck, 6020 Innsbruck, Austria; birgit.waltenberger@uibk.ac.at (B.W.); stefan.schwaiger@uibk.ac.at (S.S.); 2 Department of Pharmacognosy, University of Vienna, 1090 Vienna, Austria; liurongxia107@outlook.com (R.L.); elke.heiss@univie.ac.at (E.H.H.); verena.dirsch@univie.ac.at (V.M.D.); 3 School of Pharmacy, Yantai University, 264005 Yantai, China

**Keywords:** atherosclerosis, bitter plants, *Centaurium erythraea*, *Frasera caroliniensis*, *Gentiana lutea*, Gentianaceae, gentisin, restenosis, vascular smooth muscle cell proliferation, xanthones

## Abstract

Aberrant proliferation of vascular smooth muscle cells (VSMC) plays a major role in restenosis, the pathological renarrowing of the blood vessel lumen after surgical treatment of stenosis. Since available anti-proliferative pharmaceuticals produce unfavorable side effects, there is high demand for the identification of novel VSMC proliferation inhibitors. A natural product screening approach using a resazurin conversion assay enabled the identification of gentisin (**1**) from *Gentiana lutea* as a novel inhibitor of VSMC proliferation with an IC_50_ value of 7.84 µM. Aiming to identify further anti-proliferative compounds, 13 additional nonprenylated xanthones, isolated from different plant species, were also tested. While some compounds showed no or moderate activity at 30 µM, 1-hydroxy-2,3,4,5-tetramethoxyxanthone (**4**), swerchirin (**6**), and methylswertianin (**7**) showed IC_50_ values between 10.2 and 12.5 µM. The anti-proliferative effect of **1**, **4**, **6**, and **7** was confirmed by the quantification of DNA synthesis (BrdU incorporation) in VSMC. Cell death quantification (determined by LDH release in the culture medium) revealed that the compounds are not cytotoxic in the investigated concentration range. In conclusion, nonprenylated xanthones are identified as novel, non-toxic VSMC proliferation inhibitors, which might contribute to the development of new therapeutic applications to combat restenosis.

## 1. Introduction

Coronary artery disease is a major cause of death worldwide. It is primarily caused by atherosclerosis, a complex process that involves inflammation, deposition of lipids, smooth muscle cell proliferation, and plaque formation. This results in stenosis, the abnormal narrowing of a blood vessel caused by thickening of the arterial wall, and leads to insufficient blood flow [1]. To counteract this obstruction, surgical interventions are performed. However, a common adverse event of such surgical treatment is restenosis, the pathological recurrence of blood vessel constriction. This process is strongly associated with the excessive proliferation of vascular smooth muscle cells (VSMC) [2].

Anti-proliferate compounds, e.g., paclitaxel (taxol) and rapamycin, are used in drug-eluting stents, in order to overcome restenosis by inhibition of VSMC growth. Unfortunately, these substances produce unfavorable side effects such as impaired reendothelialization and thrombosis induction [3]. Therefore, due to the side effect profiles of available pharmaceuticals, there is great interest in the discovery of new VSMC proliferation inhibitors [2].

Plant-derived natural products are evolutionarily privileged structures which provide structural complexity and affinity for biological targets [4]. They represent an excellent basis for the search for novel biologically active compounds and the development of new therapeutic drugs [5].

The aim of our study was to find novel natural product inhibitors of VSMC proliferation. Therefore, we tested in-house available natural products using a resazurin conversion assay to assess their potential to inhibit VSMC proliferation. This approach led to the identification of a xanthone derivative from *Gentiana lutea* as a novel VSMC proliferation inhibitor. Further compounds with similar chemical structures, isolated from different plant species of the Gentianaceae family, were additionally selected for testing of their anti-proliferative activity. Measurement of DNA synthesis in VSMC by quantification of 5-bromo-2′-deoxyuridine (BrdU) incorporation into DNA was used to confirm the anti-proliferative effect of the most promising compounds. Cell death quantification (based on lactate dehydrogenase (LDH) release in the culture medium) was utilized to assess potential toxicity.

## 2. Results and Discussion

**Figure 1:**
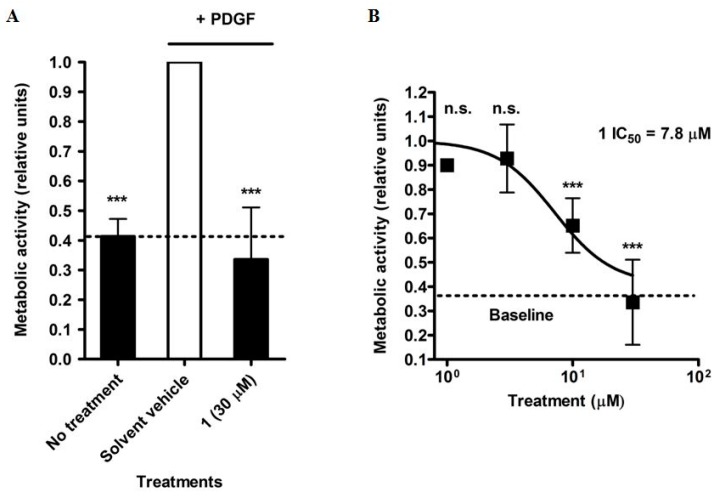
Inhibition of VSMC proliferation by gentisin (**1**), determined by the resazurin conversion method. Cells were treated with the indicated concentrations of **1** for 48 h. (**A**) represents the data obtained from VSMC treated or untreated with PDGF, as well as the inhibitory effect of 30 µM **1** on PDGF-induced VSMC proliferation; (**B**) depicts the results obtained from dose response studies (investigated concentrations: 1, 3, 10, and 30 µM) used to determine the IC_50_ of **1**. Values are presented as means ± SD, relative to the PDGF stimulated solvent vehicle control (0.1% DMSO) (*n* = 3). *** *p* < 0.001, n.s. not significant (ANOVA/Bonferroni).

With the aim to identify novel natural product inhibitors of VSMC proliferation, we tested several natural products (not shown) from our in-house compound collection on their ability to inhibit proliferation of rat aortic VSMC induced by the platelet-derived growth factor (PDGF), which is a mitogen strongly associated with aberrant proliferation in restenosis [6] and leads to excessive proliferation of VSMC. After stimulation of the VSMC for 48 h with PDGF, the total number of metabolically active cells was measured by resazurin conversion. In this way, the xanthone gentisin (1,7-dihydroxy-3-methoxyxanthone (**1**) [7]), previously isolated from the root material of *G. lutea* [8], a well-known medicinal bitter plant from the Gentianaceae family, was identified as a novel inhibitor of VSMC proliferation. At a concentration of 30 µM, the compound completely inhibited PDGF-induced proliferation to the basal level (Figure 1A). Although an anti-proliferative effect of a prenylated xanthone has been described earlier [9], it represents a novel activity for simple, nonprenylated xanthones.

**Figure 2:**
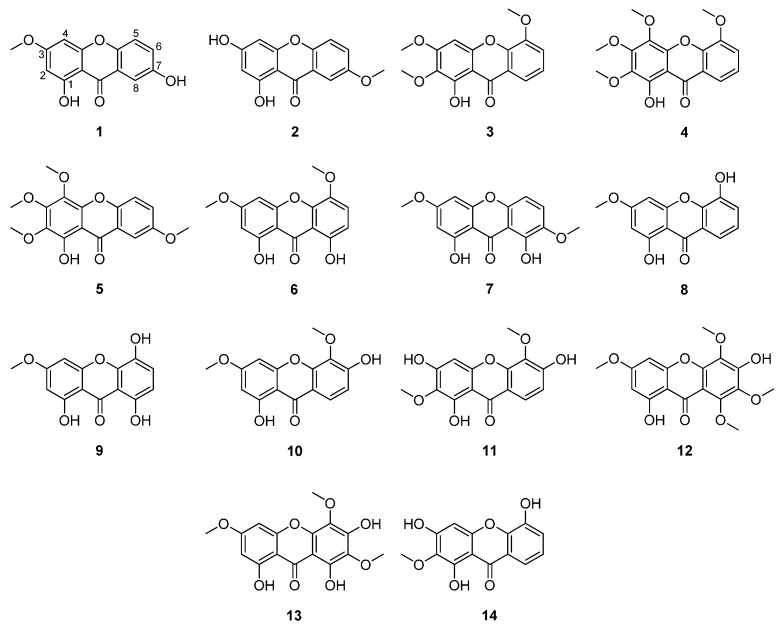
Chemical structures of xanthones **1**–**14**.

To identify further active compounds and to possibly gain knowledge about the structure activity relationship (SAR), further xanthone derivatives with structural similarity to **1**, derived from different bitter plants from the Gentianaceae family, were additionally selected for biological testing. Figure 2 depicts compound **1** as well as the additionally selected xanthones.

Isogentisin (1,3-dihydroxy-7-methoxyxanthone (**2**) [7]), the isomer of compound **1**, also previously isolated from yellow gentian root material in the same phytochemical approach [8], was one of the additionally selected analogs. Both compounds are xanthones with a simple 1,3,7-oxygenation pattern and one methoxy substituent. They differ only in the position of the methoxy substituent, which is in position 3 in **1** and in position 7 in compound **2**.

In addition, four xanthones (1-hydroxy-2,3,5-trimethoxyxanthone (**3**) [10], 1-hydroxy-2,3,4,5-tetramethoxyxanthone (**4**) [11], 1-hydroxy-2,3,4,7-tetramethoxyxanthone (**5**) [11], and 1,8-dihydroxy-3,5-dimethoxyxanthone (swerchirin) (**6**) [12]), previously derived from the roots of *Frasera caroliniensis* [13], also called American columbo, another bitter plant from the Gentianaceae family, were also selected for biological testing. These compounds also comprise the same structural skeleton like compound **1**, but with a slightly different substitution pattern. While **1** and **2** are trisubstituted xanthones, the compounds selected from *F. caroliniensis* possess a higher oxidation level. Compounds **3** and **6** are tetraoxygenated, whereby compounds **4** and **5** are pentaoxygenated xanthones. With the exception of **6**, which comprises two hydroxy groups, these compounds bear one hydroxy substituent. All other substituents are methoxy groups. In addition to the higher number of methoxy groups and total substituents, these compounds also differ in the position of the substituents.

Moreover, eight xanthone derivatives (1,8-dihydroxy-3,7-dimethoxyxanthone (methylswertianin) (**7**) [12], 1,5-dihydroxy-3-methoxyxanthone (mesuaxanthone A) (**8**) [14], 1,5,8-trihydroxy-3-methoxyxanthone (bellidifolin) (**9**) [15], 1,6-dihydroxy-3,5-dimethoxyxanthone (**10**) [16,17], 1,3,6-trihydroxy-2,5-dimethoxyxanthone (**11**) [18], 1,6-dihydroxy-3,5,7,8-tetramethoxyxanthone (**12**) [16,19], 1,6,8-trihydroxy-3,5,7-trimethoxyxanthone (**13**) [16], and 1,3,5-trihydroxy-2-methoxyxanthone (tovopyrifolin C) (**14**) [20], Figure 2) previously isolated from the aerial parts of *Centaurium erythraea* [13], well known as small centaury, were also selected for biological testing. Compound **6**, isolated from *F. caroliniensis*, is also present in *C. erythraea* [13]. These xanthone derivatives are structurally similar to compound **1**, but with a slightly different substitution pattern. They bear three to six substituents, all hydroxy or methoxy groups, in various positions.

Whereas some of these 13 additional xanthones showed no or only weak activity at a concentration of 30 µM, five of them (*i.e.*, **2**, **4**, **6**, **7**, and **8**) inhibited PDGF-stimulated proliferation of VSMC with residual activity values of less than 0.75 relative units (RU) at a concentration of 30 µM (Table 1). These five compounds, together with **1**, which reduced VSMC proliferation to 0.34 RU at 30 µM, were thus analyzed in more detail in concentration response studies using the resazurin conversion assay. All six compounds inhibited VSMC proliferation in a concentration-dependent manner. Compounds **4**, **6**, and **7** showed IC_50_ values of 11.2, 12.5, and 10.2 µM, while **2** and **8** were less active in this test system with IC_50_ values of 27.0 and 29.5 µM, respectively (Table 1). Gentisin (**1**) was the most active compound. It exhibited a concentration-dependent inhibition of VSMC proliferation with an IC_50_ value of 7.84 µM (Figure 1B). For comparison, we have also determined the effect of the clinically used VSMC proliferation inhibitor taxol, which displayed an IC_50_ value of 0.1 µM [21].

**Table 1:** Inhibition of VSMC proliferation by compounds **1**–**14** in the resazurin conversion and in the BrdU incorporation assays. Data (means ± SD, *n* = 3) are presented as relative units (RU) in comparison to the control (cells treated with PDGF in the presence of 0.1% DMSO vehicle; 1.00 RU) or IC_50_ values.

Compound Number	Compound Name	Inhibition of VSMC Proliferation		
		Resazurin Conversion Assay		BrdU Assay
		at 30 µM (Relative to Solvent vehicle = 1.00, RU)	IC_50_ (µM)	IC_50_ (µM)
**1**	1,7-diOH-3-MeO-xanthone (gentisin) ^a^	0.335 ± 0.175	7.84	5.74
**2**	1,3-diOH-7-MeO-xanthone (isogentisin) ^a^	0.593 ± 0.084	27.0	n.d. ^d^
**3**	1-OH-2,3,5-triMeO-xanthone ^b^	0.832 ± 0.104	n.d.	n.d.
**4**	1-OH-2,3,4,5-tetraMeO-xanthone ^b^	0.216 ± 0.058	11.2	24.5
**5**	1-OH-2,3,4,7-tetraMeO-xanthone ^b^	0.754 ± 0.014	n.d.	n.d.
**6**	1,8-diOH-3,5-diMeO-xanthone (swerchirin) ^b, c^	0.514 ± 0.067	12.5	7.37
**7**	1,8-diOH-3,7-diMeO-xanthone (methylswertianin) ^c^	0.002 ± 0.014	10.2	7.53
**8**	1,5-diOH-3-MeO-xanthone (mesuaxanthone A) ^c^	0.713 ± 0.061	29.5	n.d.
**9**	1,5,8-triOH-3-MeO-xanthone (bellidifolin) ^c^	0.783 ± 0.031	n.d.	n.d.
**10**	1,6-diOH-3,5-diMeO-xanthone ^c^	0.987 ± 0.025	n.d.	n.d.
**11**	1,3,6-triOH-2,5-diMeO-xanthone ^c^	0.942 ± 0.042	n.d.	n.d.
**12**	1,6-diOH-3,5,7,8-tetraMeO-xanthone ^c^	0.862 ± 0.068	n.d.	n.d.
**13**	1,6,8-triOH-3,5,7-triMeO-xanthone ^c^	0.846 ± 0.096	n.d.	n.d.
**14**	1,3,5-triOH-2-MeO-xanthone (tovopyrifolin C) ^c^	0.862 ± 0.104	n.d.	n.d.

^a^ constituent of *G. lutea*; ^b^ constituent of *F. caroliniensis*; ^c^ constituent of *C. erythraea*; ^d^ n.d., not determined.

Since the resazurin conversion assay is based on the ability of viable cells to transform resazurin into the fluorescent compound resorufin, there is a good correlation between the measured fluorescence and the number of living cells. However, in this model, substances might modulate the cellular metabolic capacity or directly interfere with the redox-dependent resazurin to resorufin conversion, thus leading to false activity estimation.

**Figure 3:**
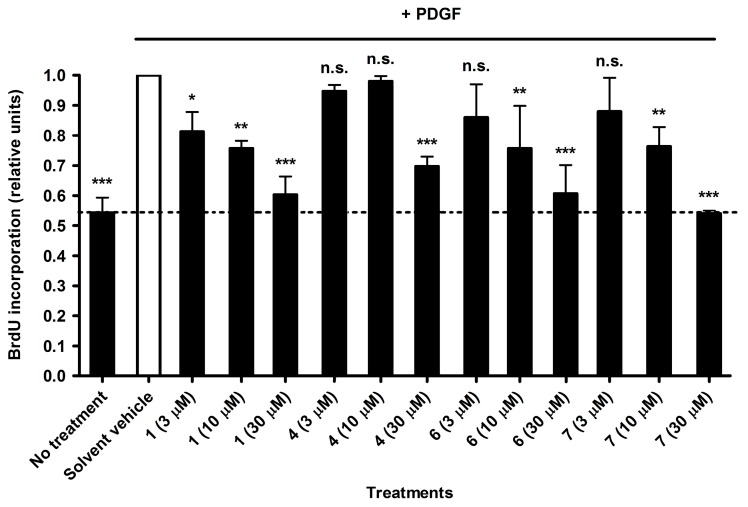
Inhibition of VSMC proliferation by compounds **1**, **4**, **6**, and **7** measured with the BrdU assay. Values are presented as means ± SD, relative to the PDGF stimulated solvent vehicle control (0.1% DMSO) (*n* = 3). *** *p* < 0.001, ** *p* < 0.01, * *p* < 0.05, n.s. not significant (ANOVA/Bonferroni).

Thus, in order to confirm the anti-proliferative activity of the four most active xanthones **1**, **4**, **6**, and **7**, which exhibited IC_50_ values between 7.84 and 12.5 µM in the resazurin conversion assay, they were additionally investigated using the BrdU incorporation assay. In this assay, the DNA synthesis in dividing VSMC is measured by quantification of BrdU incorporation into newly synthesized DNA. All four compounds showed activity in a similar range as in the resazurine model. Moreover, in all four cases, higher compound concentration led to stronger inhibition of VSMC proliferation. Thus, a concentration-dependent inhibition was observed. In particular, compounds **1**, **4**, **6**, and **7** inhibited DNA synthesis with IC_50_ values of 5.74, 24.5, 7.37, and 7.53 µM, respectively (Figure 3, Table 1). For comparison, we have also determined the effect of the cyclin-dependent kinase (CDK) inhibitor roscovitine, which displayed an IC_50_ value of 16.9 µM [22].

**Figure 4:**
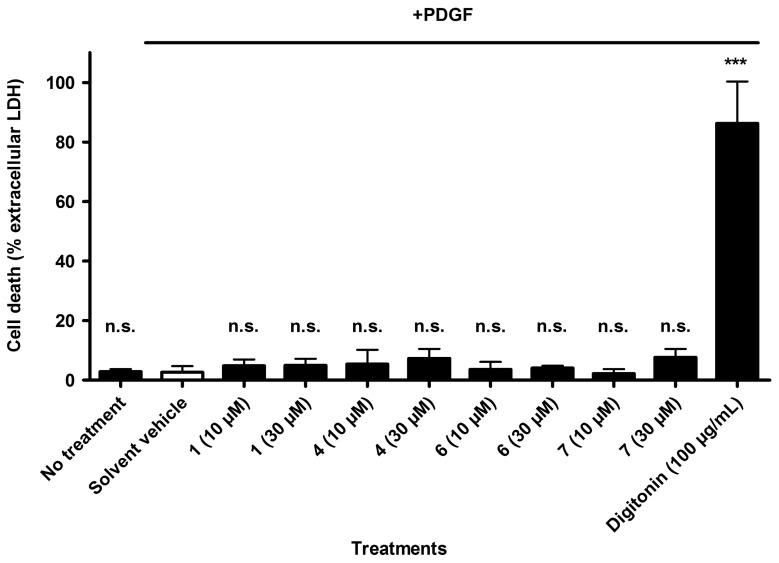
Determination of potential cytotoxic effects of compounds **1**, **4**, **6**, and **7**. Digitonin (100 µg/mL, corresponding to 81 µM) was used as positive control, vehicle (0.1% DMSO) with and without PDGF was used as negative control. Values are presented as means ± SD (*n* = 3). *** *p* < 0.001, n.s. not significant (ANOVA/Bonferroni).

To ensure that the observed inhibitory effect on VSMC proliferation is not due to cytotoxicity, the four most active compounds were additionally analyzed for potential cytotoxic effects. Loss of VSMC membrane integrity as a sign for cell death was quantified by measuring the release of the soluble cytosolic protein LDH. This assay revealed that the compounds are not cytotoxic in the investigated concentration range up to 30 µM (Figure 4).

While an anti-proliferative effect of cudratricusxanthone A, an isoprenylated xanthone from the root bark of *Cudrania tricuspidata*, was described by Kim *et al.* [9], this is the first report of nonprenylated xanthones as inhibitors of VSMC proliferation.

In the present study, 14 xanthone derivatives were investigated on their anti-proliferative effects in rat aortic VSMC. Although they are structurally closely related, they demonstrated very different activities. Gentisin (**1**) turned out to be the most potent compound, but also **4**, **6**, and **7** showed potent inhibitory activity, whereas **2** and **8** were moderately active and compounds **3**, **5**, and **9**–**14** were weakly active or inactive. However, based on these data, no clear conclusions about the SAR of these compounds could be drawn, since there is no clear relation between the activity of the xanthones and their substitution pattern. Therefore, further studies including the investigation of additional xanthone derivatives are necessary to gain better knowledge about the SAR of simple, oxygenated xanthones. Moreover, VSMC proliferation is a complex phenotypic effect that may be a result of various interactions between a small molecule and regulatory proteins whereby the modification of a small molecule might enhance interaction with one relevant protein but simultaneously weaken the interaction with other relevant protein(s). Therefore, the lack of a clear SAR in this study might also be due to a complex mechanism of action. Thus, to reveal a clear SAR, more detailed studies on the molecular mechanism of action of the xanthones are required.

The four most active xanthones are constituents of *G. lutea* (compound **1**), *F. caroliniensis* (compounds **4** and **6**), and *C. erythraea* (compounds **6** and **7**). However, the most potent xanthone derivative **1**, gentisin, was derived from *G. lutea*, together with its moderately active isomer **2**. Therefore, in order to determine whether the anti-proliferative activity of these compounds affects the activity of the crude extract, we additionally investigated the crude methanol extract of *G. lutea* roots [8], from which the compounds were previously isolated and which had been stored at −80 °C. The extract was tested in the resazurin conversion assay at a concentration of 30 µg/mL, but with inhibition of VSMC proliferation to 0.846 ± 0.020 RU, the observed anti-proliferative activity was only weak. In a previous work [23] we have determined the phytochemical profile of the *G. lutea* root methanol extract and quantified the main constituents in extracts of several commercially available *G. lutea* samples. The most dominant compound with a concentration of up to 9.53% was the bitter tasting secoiridoid glycoside gentiopicroside, followed by loganic acid, swertiamarin, and the glycosides of **1** and **2**. The aglycones **1** and **2** were present in much lower concentrations. While the concentration of the moderately active **2** was determined to be 0.04%–0.11%, the most active compound **1** was present in a concentration of only 0.02%–0.07%. The low concentration of the active compounds is likely the reason for the weak anti-proliferative activity of the crude extract.

Kesavan *et al.* [24] reported the inhibition of PDGF induced proliferation of rat aortic smooth muscle cells by a water extract of *G. lutea* roots, however, at a much higher concentration (1 mg/mL). Furthermore, they identified the flavonoid isovitexin as an inhibitor of VSMC proliferation. Akileshwari *et al.* [25] suggested the presence of this compound in methanol and water extracts of *G. lutea* roots due to the monitored *m*/*z* of 433.8520 [M + H]^+^ in a MALDI-TOF analysis. This is not in accordance with a study by Kusar *et al.* [26], who investigated the extraction of isovitexin, among other compounds, from yellow gentian leaves and roots using methanol, ethanol, and/or water as extraction solvents. While isovitexin was present in the leaf extracts, it could not be detected by HPLC-UV in comparison with reference compounds in any of the root extracts. This is in agreement with our results. We could also not detect isovitexin in our methanolic *G. lutea* root extract, while the xanthones **1** and **2** were present in minor amounts.

However, the results show that the bioactivity-guided approach often followed is not always the best choice. An extract possessing only weak activity is usually not investigated further. In this case, starting with crude extracts and only continuing with highly potent extracts would have led to the missing out of an interesting bioactivity. Instead, direct investigation of isolated natural products as an alternative approach led to the identification of novel inhibitors of VSMC proliferation. Especially for applications for which extracts cannot be considered due to their overly complex composition, as is the case for a potential topical application in a stent, direct testing of isolated natural products, enabling the quick identification of pure compounds as active agents, is meaningful. Moreover, the bioactivity of compounds that are present only in trace quantities in plant extracts, can also be identified in this way.

In this study, using a direct testing of natural products strategy, we identified gentisin (**1**) as a novel inhibitor of VSMC proliferation. Pharmacological investigation of additional xanthones with related chemical structures led to the identification of further active compounds. The activity of the most potent compounds was confirmed by inhibition of VSMC proliferation in a BrdU assay and it was shown that the compounds are not cytotoxic. Another advantage of these compounds is their high chemical stability, which could be a benefit in the manufacturing process. Stability studies conducted according to the ICH-guidelines (www.ich.org) revealed that they are stable under long-term, accelerated, and refrigerated conditions [13].

## 3. Experimental Section

### 3.1. Chemicals and Reagents

Growth media, serum, and cell culture supplements were purchased from Lonza (Braine-L’Alleud, Belgium). The chemoluminescent BrdU cell proliferation kit was obtained from Roche Diagnostics (Vienna, Austria). All other reagents used were of analytical grade and obtained from Sigma-Aldrich (Vienna, Austria).

### 3.2. Cell Culture

Rat aortic VSMC were acquired from Lonza and grown in Dulbecco’s modified essential medium (DMEM)–F12 (1:1) supplemented with 20% fetal calf serum (FCS) and gentamycin. For all experiments, cells were seeded in 96-well plates (5 × 10^3^ cells/well) and incubated for 24 h. Subsequently, cells were serum-starved for another 24 h to make them quiescent.

### 3.3. Resazurin Conversion Assay

Quiescent VSMC were pre-treated with the studied test compounds, *G. lutea* root extract, or vehicle (0.1% DMSO) for 30 min and then stimulated with PDGF-BB (20 ng/mL) for 48 h. The number of metabolically active VSMC was measured by resazurin conversion [27,28]. Therefore, after washing the cells with phosphate-buffered saline (PBS), they were incubated in serum-free medium containing resazurin (10 µg/mL) for 2 h. Measurement of total metabolic activity was performed by monitoring the increase in fluorescence at 590 nm using an excitation wavelength of 535 nm in a 96-well plate reader (Tecan GENios Pro, Grödig, Austria). The assay was validated using the VSMC proliferation inhibitor taxol (IC_50_ = 0.1 µM [21]).

### 3.4. 5-Bromo-2′-deoxyuridine (BrdU) Incorporation Assay

Quiescent VSMC were pre-treated with test compounds or vehicle (0.1% DMSO) for 30 min and then stimulated with PDGF-BB (20 ng/mL) for 2 h. Subsequently, BrdU was added to estimate *de novo* DNA synthesis in VSMC [29,30]. After 22 h, the BrdU incorporation was determined according to the instructions of the manufacturer (Roche Diagnostics, Vienna, Austria). The assay was validated using the CDK inhibitor roscovitine (IC_50_ = 16.9 µM [22]).

### 3.5. Assessment of Cytotoxicity

Quiescent VSMC were pre-treated with test compounds, positive control (digitonin 100 µg/mL, corresponding to 81 µM), or vehicle (0.1% DMSO) for 30 min and then stimulated with PDGF-BB (20 ng/mL) for 24 h. Loss of cell membrane integrity as an indicator for cell death was quantified by measuring the release of LDH [31,32]. Therefore, the LDH activity of the cell supernatant was determined. To estimate total LDH, identically treated samples were incubated in the presence of 1% Triton X-100 for 45 min. The activity of released and total LDH enzyme activity was measured for 30 min in the dark in presence of lactate (4.5 mg/mL), NAD^+^ (0.56 mg/mL), diaphorase (1.69 U/mL), 0.004% (*w*/*v*) BSA, 0.15% (*w*/*v*) sucrose, and 0.5 mM 2-p-iodophenyl-3-nitrophenyl tetrazolium chloride (INT). The enzyme reaction was stopped by oxymate (1.78 mg/mL) and the absorbance was read at 490 nm. The percentage of extracellular LDH enzyme activity was calculated to estimate potential effects of the samples on cell viability.

### 3.6. Statistical Analysis

Statistical analysis was conducted by ANOVA/Bonferroni test using the software GraphPad PRISM, version 4.03 (GraphPad Software, La Jolla, CA, USA). The number of experiments is provided in the figure legends. A probability value *p* < 0.05 was considered significant.

## 4. Conclusions

This study shows that the direct testing of already isolated natural products is a valuable strategy to find new bioactivities. In this study, it enabled the identification of nonprenylated xanthones of high chemical stability as novel, non-toxic inhibitors of VSMC proliferation, which might be a starting point for the development of new therapeutic applications to combat restenosis.
